# Unpacking extracellular vesicles: RNA cargo loading and function

**DOI:** 10.1002/jex2.40

**Published:** 2022-05-02

**Authors:** Elizabeth R. Dellar, Claire Hill, Genevieve E. Melling, David R.F Carter, Luis Alberto Baena‐Lopez

**Affiliations:** ^1^ Department of Biological and Medical Sciences Oxford Brookes University Gipsy Lane Oxford UK; ^2^ Sir William Dunn School of Pathology University of Oxford South Parks Road Oxford UK; ^3^ Nuffield Department of Clinical Neurosciences John Radcliffe Hospital University of Oxford Oxford UK; ^4^ Institute of Clinical Sciences School of Biomedical Sciences College of Medical and Dental Sciences University of Birmingham Edgbaston Birmingham UK

**Keywords:** delivery, EVs, extracellular vesicles, intercellular communication, loading, motifs, RBPs, RNA loading, RNA, RNA‐binding proteins, zipcodes

## Abstract

Extracellular vesicles (EVs) are a heterogeneous group of membrane‐enclosed structures produced by prokaryotic and eukaryotic cells. EVs carry a range of biological cargoes, including RNA, protein, and lipids, which may have both metabolic significance and signalling potential. EV release has been suggested to play a critical role in maintaining intracellular homeostasis by eliminating unnecessary biological material from EV producing cells, and as a delivery system to enable cellular communication between both neighbouring and distant cells without physical contact. In this review, we give an overview of what is known about the relative enrichment of the different types of RNA that have been associated with EVs in the most recent research efforts. We then examine the selective and non‐selective incorporation of these different RNA biotypes into EVs, the molecular systems of RNA sorting into EVs that have been elucidated so far, and the role of this process in EV‐producing cells. Finally, we also discuss the model systems providing evidence for EV‐mediated delivery of RNA to recipient cells, and the implications of this evidence for the relevance of this RNA delivery process in both physiological and pathological scenarios.

## 1 INTRODUCTION

Extracellular vesicles (EVs) are small membrane‐bound particles that are released by cells into the extracellular environment. First described as lipid‐rich ‘platelet dust’ in 1967, both plasma‐membrane‐shed vesicles, and extracellularly released internal vesicles were discovered in the 1980s (Couch et al., 2021; Harding et al., 1984; Pan & Johnstone, 1983; Trams et al., 1981; Wolf, 1967). These vesicles were thought to be a mechanism for shedding of the transferrin receptor from reticulocytes, leading to the belief that EVs function primarily as a means of cellular waste disposal. Later work suggested a signalling function for EVs, with the demonstration that they contribute to initiation or amplification of immune response via antigenic presentation (Raposo et al., 1996; Théry et al., 2002). Between 2006 and 2008, it was discovered that EVs may contain coding messenger RNAs (mRNAs) and microRNAs (miRNAs), thus greatly fuelling the study of EVs and their cargo (Baj‐Krzyworzeka et al., 2006; Ratajczak et al., 2006; Skog et al., 2008; Valadi et al., 2007). EV RNAs have been identified as a source of novel disease biomarkers, for example, the detection of mutated *EGFRvIII* within EVs purified from glioblastoma patient serum has the potential to advance diagnostic capabilities and aid mutation‐specific treatment planning (Skog et al., 2008). Beyond this diagnostic use, mRNAs loaded in EVs were also shown to be translatable in recipient cells in vitro, leading to the notion of EVs acting as an intercellular messaging system (Valadi et al., 2007). RNAs have been shown to be present in bacterial (Sjöström et al., 2015), fungal (Da Silva et al., 2015), insect (Lefebvre et al., 2016), parasitic (Herron et al., 2022), and plant EVs (Baldrich et al., 2019), as well as in mammalian EVs, suggesting that this messaging system is a conserved function. Indeed, further work has suggested that functional effects of EVs on the cellular proliferation and viability of recipient cell populations is mediated specifically by the loaded RNA, indicating a wide role for EV loaded RNA in both physiological and pathological scenarios (Eldh et al., 2010; Ratajczak et al., 2006).

**FIGURE 1:**
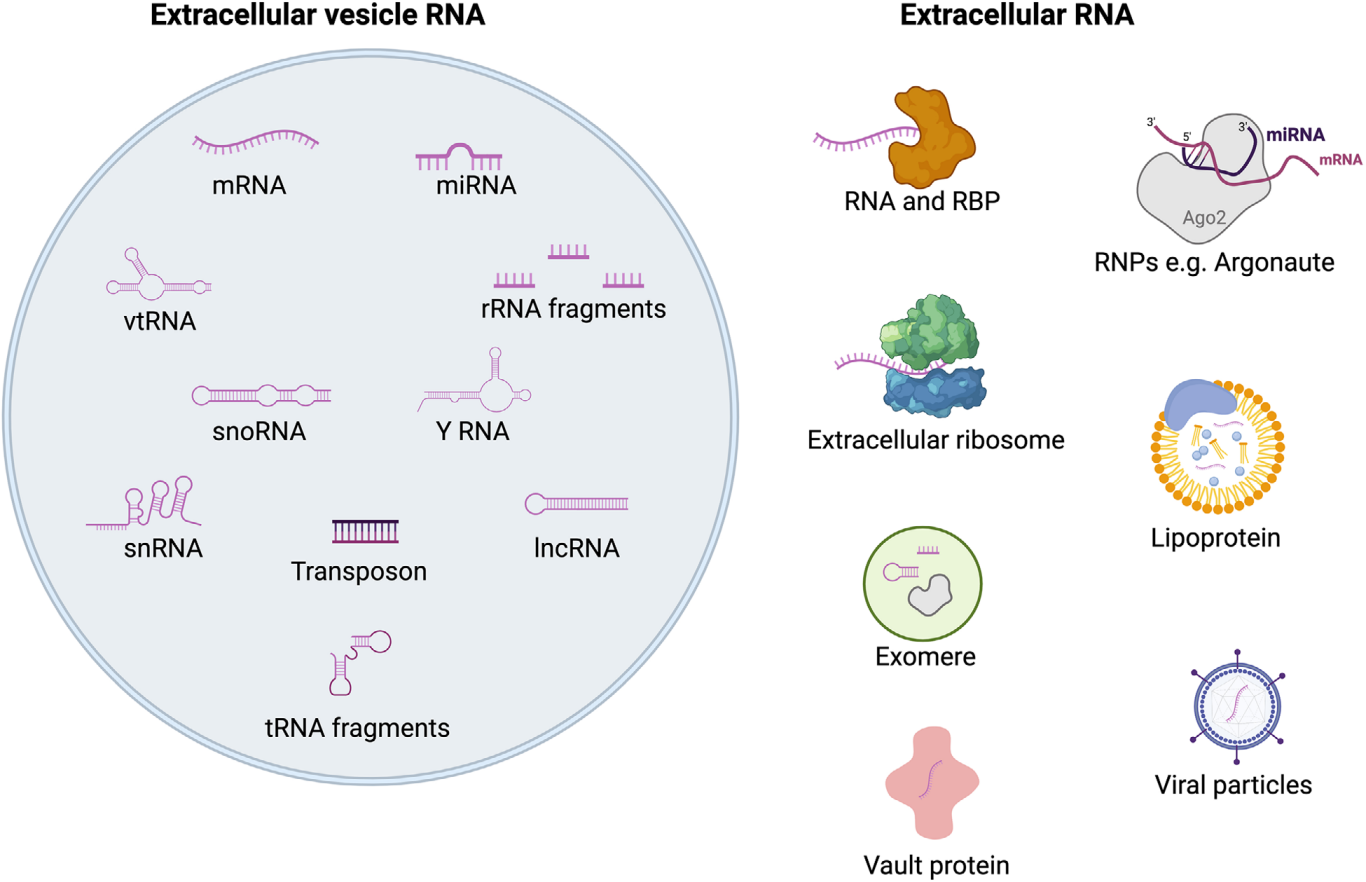
Types of extracellular vesicle (EV) and extracellular RNA. Schematic diagram to represent the types of RNA found associated with extracellular vesicles (EVs), along with other co‐isolated extracellular RNAs. A number of extracellular complexes, such as RNAs and RNA binding proteins (RBPs), RNAs and Argonautes, or vault particles containing vault RNA (vtRNA) can also be found within/associated with EVs. Argonautes have also been reported in non‐membrane‐bound exomeres (Q. Zhang et al., 2019; H. Zhang et al., 2018). RBP‐RNA complexes can contain different RNAs such as messenger RNA (mRNA) or microRNA (miRNA) molecules. Although only ribosomal RNA (rRNA) and transfer RNA (tRNA) fragments are illustrated within EVs, other RNA fragments such as mRNA fragments, may also be present

EVs are typically classified into three major types depending on distinct biogenesis pathways (Raposo & Stoorvogel, 2013). Firstly, exosomes, 50–150 nm vesicles deriving from intraluminal vesicles (ILVs) in multivesicular bodies (MVBs), requiring microtubular transport of the MVB to the plasma membrane to allow fusion and extracellular release of the ILVs, an alternative to degradation of ILV contents by MVB‐lysosome fusion. Secondly, microvesicles (MVs), otherwise known as ectosomes, with a variable size range of 100–1000 nm diameter, bud directly from the cell plasma membrane. Apoptotic bodies (ABs) have an even broader size range than exosomes and MVs, at 50–5000 nm, and are formed during programmed cell death (Poon et al., 2014). In addition, overlapping sub‐types such as oncosomes, large (1–10 μm) but non‐apoptotic, cancer cell‐derived EVs that bud from the plasma membrane have also been defined (Di Vizio et al., 2009, 2012). Several groups have described the existence of additional non‐vesicular nanoparticles which have overlapping characteristics; 35–50 nm ‘exomeres’, notably enriched in Argonaute proteins (H. Zhang et al., 2018; Q. Zhang et al., 2019), and other ribonucleoprotein particles containing components similar to those reported for EVs (Turchinovich et al., 2011; Wei et al., 2017). However, other evidence indicates that the Argonaute proteins, and associated bound miRNAs, are present within the EVs (Barman et al., 2020; Clancy et al., 2019; Mckenzie et al., 2016; Melo et al., 2014). Finally, there is also growing appreciation of the existence of other extracellular RNA that is not vesicle‐associated, including extracellular ribosomes and mRNA, which may contribute some of the RNA and functions previously ascribed to vesicular RNA (Figure 1) (Tosar et al., 2021). Thus, there is a high degree of overlap between all types of nanoparticles, so defining specific sub‐type markers is a key area of study (Jeppesen et al., 2019; Kowal et al., 2016; Mathieu et al., 2021; Willms et al., 2016).

Widespread employment of RNA sequencing has demonstrated that EVs/EV‐like particles can contain many RNA biotypes, including non‐coding RNA types such as snoRNA, snRNA, lncRNA, vault RNA, Y‐RNA, tRNA and rRNA, or fragments thereof (Figure 1) (Jenjaroenpun et al., 2013; Kogure et al., 2013; Nolte‐’T Hoen et al., 2012). Whilst all these RNA subtypes have been identified in EVs in some studies, technical differences between these studies have resulted in often conflicting data on deeper investigation, as a result of differences in cell types, purification protocols and EV sub‐populations under study (Mateescu et al., 2017). In particular, different RNA sequencing technologies predetermine which RNAs can be discovered, for example, differential size selection in small RNA sequencing, poly(A) selection of mRNA, or ribo‐depletion. Since such sequencing experiments are also carried out on bulk preparations of EVs, they give no information on the heterogeneity of RNA distribution between single EVs, necessary to understanding of their function. We are thus far from a clear understanding of how they become loaded during EV biogenesis, and whether the specific RNA cargo is of particular importance to the either producing or receiving cells.

## 2 RNA BIOTYPES PRESENT WITHIN EVS

Intact **mRNAs** are long RNAs of between ∼150 and 60,000 nt in length that code for protein production. Within their DNA‐encoded sequence, they contain coding exons (CDS) interspersed within non‐coding introns, preceded by a 5′ untranslated region (5′UTR) and followed by a 3′untranslated region (3′UTR), both of which are involved in regulating stability, localisation, and translation of the mRNA. Some studies have detected full‐length long mRNAs in EVs, of up to 5000 bp in length (by comparison of qPCR Ct values using oligo‐dT and random primers, or by using long amplicons) (Enderle et al., 2015; Matsuno et al., 2019), whilst others have been unable to detect full‐length RNAs longer than 1000 bp (Hinger et al., 2018; Wei et al., 2017). The presence of intact mRNA may also be inferred, but not proved, from full‐length coverage in RNA sequencing studies. However, since the majority of RNA in EV samples lies between 25 and 700 nt in bioanalyser traces, this has indicated a high prevalence of smaller RNAs and/or fragments of longer ones. Mosbach et al. (2021) investigate the length dependence, and find that, for polymerase‐III‐encoded transcripts, there is an inverse relationship between RNA length and EV/cell abundance ratio when looking at 80–680 nt transcripts. Several studies have also indicated that UTR regions, particularly 3′UTRs, are overrepresented in EVs relative to the coding region (Batagov & Kurochkin, 2013; Nolte‐’T Hoen et al., 2012; Pérez‐Boza et al., 2018; Wei et al., 2017; Zand Karimi et al., 2022). These fragments may represent preferentially stable products of the degradation of full‐length mRNAs which are targeted to EVs for disposal, since, for example, nonsense‐mediated decay has been found to be less efficient at the 3′ end of mRNAs (Gout et al., 2017). Alternatively, since differential expression of 3′UTRs relative to the coding region has been documented in several studies in cells (Kocabas et al., 2015; Mercer et al., 2011; Sudmant et al., 2018), it is also possible that isolated 3′UTRs may be specifically incorporated into the EVs and act through regulation of gene expression, functioning as a molecular ‘sponge’ for regulatory miRNA and translation factors (Wei et al., 2017).

**Circular RNAs (circRNAs)** are single‐stranded RNAs that typically form through alternative splicing of mRNAs, via a back‐splicing mechanism that joins together the 5′ and 3′ ends of exons or introns (Patop et al., 2019). As such, mRNAs exist in both their linear and circular forms, but with the latter highly resistant to exonuclease‐mediated decay, so are therefore highly stable. Several studies have identified some mRNAs of which the circular form may be enriched within EVs, via either RT‐qPCR or RNA sequencing approaches (Dou et al., 2016; Lasda & Parker, 2016; Li et al., 2019; Preußer et al., 2018). Preußer et al. (2018) demonstrate that circRNAs GSE1 and NRIP1 cofractionate with the EV marker CD63 using sucrose‐density gradient centrifugation for EV purification, supporting their presence within EVs rather than co‐isolation. CircRNAs are poorly understood, but may be translated or, like mRNA fragments, function as ‘sponges’ for miRNA and proteins, or scaffolds for their transport (Patop et al., 2019).

**MiRNAs** are small ∼22 nt single‐stranded RNAs which are important in the regulation of mRNA expression, largely through interaction with 3′UTRs to cause translational repression or degradation (Bartel, 2004). miRNAs have been one of the most heavily studied of RNA cargos in EVs, likely because they are easier to identify, easier to delineate a mechanism of action, and have great biomedical interest, both as biomarkers of disease and from the perspective of RNA‐mediated therapies (Pérez‐Boza et al., 2018). In small RNA sequencing, miRNA has been reported to make up anywhere from <1% to 30% of total reads (Baglio et al., 2015; Chiou et al., 2018; Koppers‐Lalic et al., 2014; Nolte‐’T Hoen et al., 2012; Tosar et al., 2015). These differences may be reflective of methodological artefacts in some studies, with both bovine serum contaminants (even when media is EV‐depleted) and co‐purified extracellular ribonucleoprotein particles, such as AGO2 and high‐density lipoproteins, found to make significant contributions to total extracellular miRNA (Arroyo et al., 2011; Turchinovich et al., 2011; Vickers et al., 2011; Wei et al., 2016).

**Transfer RNAs (tRNA**) are highly structured 76–90 nt RNAs which function as adaptor molecules in translation, with a 3 nt anticodon loop for mRNA recognition and an amino acid attachment site (Goodenbour & Pan, 2006). In EVs, tRNAs have been reported to be both abundant as a percentage of total RNA, constituting up to 95% of small RNA in syncytiotrophoblast EVs, and enriched relative to cells, although again with a high abundance in free ribonucleoprotein particles (Baglio et al., 2015; Cooke et al., 2019; Tosar et al., 2015). In‐depth study of tRNA has also demonstrated the presence of fragmented tRNA, with a greater prevalence of 5′ regions relative to 3′ halves (Chiou et al., 2018; Cooke et al., 2019; Nolte‐’T Hoen et al., 2012; Wei et al., 2017).

**Small nucleolar RNAs (snoRNAs)** are stable species of around 60–300 nt in length which function in the modification or cleavage of ribosomal RNA and small nuclear RNA (Bratkovič et al., 2020). SnoRNAs have been identified in small RNA sequencing studies of EVs, generally at low abundance and depleted in EVs relative to cells (Baglio et al., 2015; Chiou et al., 2018; Driedonks et al., 2018; Lässer et al., 2017; Nolte‐’T Hoen et al., 2012; Tosar et al., 2015; Wei et al., 2017). However, James et al. (2021) found that some specific snoRNAs were highly enriched in EVs of human‐induced pluripotent stem cell cardiomyocytes, with specific alterations also seen upon electrical stimulation, but only when derived from hypertrophic cardiomyopathy patients. Additionally, snoRNA abundance was similar in human HepG2 EVs and cells when using total rather than small RNA sequencing, whilst appearing enriched in *Drosophila* cells (Lefebvre et al., 2016). snoRNAs, at 60–300 nt, lie between the boundary of small and long RNA sequencing techniques, so these differences highlight the strong influence of sequencing technology on the conclusions drawn.

**Small nuclear RNAs (snRNAs)** are ∼150 nt nuclear RNAs which associate with Sm proteins to form small nuclear ribonucleoproteins, together forming the spliceosome complex which, in concert with other protein factors, functions in mRNA splicing (Matera et al., 2007). snRNAs have also been identified in small RNA sequencing of EVs, although reported in different studies to be of similar abundance, enriched, or depleted in EVs relative to cells (Chiou et al., 2018; Driedonks et al., 2018; Lässer et al., 2017; Nolte‐’T Hoen et al., 2012). In total RNA sequencing, snRNA appears to be enriched in both human and *Drosophila* cell line EVs (Lefebvre et al., 2016).

**Long non‐coding RNAs (lncRNAs)** are >200 nt RNAs which do not code for protein but are instead associated with a range of regulatory functions, including direct or indirect transcriptional regulation, binding of miRNAs, and mRNA stability (Fatica & Bozzoni, 2014). Enrichment of lncRNAs in EVs has been investigated by several groups, who have demonstrated their involvement in mediating cell proliferation, angiogenesis and resistance to chemotherapeutic drugs in recipient cells (Gezer et al., 2014; Hewson et al., 2016; Hinger et al., 2018; Kogure et al., 2013; K. Takahashi et al., 2014).

Other non‐coding RNAs of a range of lengths have also been found to be enriched in EVs. ***7SL*,** a 300 nt RNA which forms a ribonucleoprotein complex that is involved in targeting proteins to the endoplasmic reticulum (Nabet et al., 2017; Nolte‐’T Hoen et al., 2012; Tosar et al., 2020). **Y‐RNAs,** ∼100 nt RNAs that form a ribonucleoprotein complex with the Ro60 autoantigen, functioning in the recognition, refolding and decay of variant and misfolded RNAs, such as U2 snRNA and pre‐5S ribosomal RNA (Cambier et al., 2017; X. Chen et al., 2003; Fuchs et al., 2006; Nolte‐’T Hoen et al., 2012; Tosar et al., 2015; Van Balkom et al., 2015; Wei et al., 2017). **Vault RNAs**, 86–140 nt RNAs that associate with ribonucleoprotein complexes containing major vault protein play a role in nucleocytoplasmic and cytoskeleton transport, although their function has been poorly characterised (Nolte‐’T Hoen et al., 2012; Van Balkom et al., 2015). However, alternative studies have reported vault RNAs to be absent in mammalian EVs (Jeppesen et al., 2019), whilst it has been suggested that only specific fragments of *7SL*, Y‐RNA and vault RNAs are found in EVs (Nolte‐’T Hoen et al., 2012).

**Ribosomal RNA (rRNA)** is formed of two subunits, large and small, which in mammals are made up of four RNAs, 28S, 18S, and 5.8S transcribed as a single unit, and 5S independently (Mullineux & Lafontaine, 2012). Since rRNA is generally considered to make up approximately 80% of total cell RNA, many studies opt for either small RNA sequencing, or preparation of poly(A)‐selected or ribo‐depleted total RNA libraries, in order to achieve high sequencing depth (M. Chen et al., 2016; Cherlin et al., 2020; Statello et al., 2018). Apoptotic bodies and large MVs, on the basis of bioanalyser profiles, appear to contain some intact rRNA (Crescitelli et al., 2013; Lässer et al., 2017; Wei et al., 2017) which is absent in small EVs (Bellingham et al., 2012; Lässer et al., 2011; Nolte‐’T Hoen et al., 2012; Valadi et al., 2007). However, many RNA sequencing studies have shown that, even in small EVs, ribosomal fragments are highly abundant (Berardocco et al., 2017; Jenjaroenpun et al., 2013; Miranda et al., 2014; Sork et al., 2018; Wei et al., 2017).

**Retrotransposons** are mobile genetic elements which replicate via an RNA intermediate, and have been found to be enriched in EVs in multiple studies (Ashley et al., 2018; Balaj et al., 2011; Evdokimova et al., 2019; Hardy et al., 2019; Kawamura et al., 2019). Evdokimova et al. (2019) further found that the induction of oxidative stress resulted in EV enrichment of Long Terminal Repeats, Human endogenous retroviruses and long interspersed nuclear elements, above the EV enrichment seen in normal conditions. Some evidence suggests that increases in the abundance of these RNAs are linked to cancers, brain tumour cell lines and Ewing sarcoma (Balaj et al., 2011; Evdokimova et al., 2019). Other RNAs of retroviral origin, the long non‐coding RNA VL40 in mouse dendritic cells (Barrios et al., 2021), and the neuronal mRNAs Arc and Arc1 in mammals and *Drosophila*, respectively, have also been found to be enriched within EVs (Ashley et al., 2018; Pastuzyn et al., 2018).

An important caveat to note is that with improving knowledge, the presence of co‐isolated non‐vesicular RNA has become clear (Figure 1). Increasingly, RNase protection assays—comparing the abundance of RNAse treated EV preparations with and without pre‐treatment with detergent and/or proteases—are being used to determine if different previously described ‘EV‐RNAs’ are truly vesicular. Several studies have used this approach to demonstrate miRNA, snoRNA and CRISPR/Cas9 guide RNA to be located solely within the vesicular lumen (De Jong et al., 2020; Rimer et al., 2018; Temoche‐Diaz et al., 2019). However, other studies have indicated that some is present on the outside of the vesicular membrane (Chiou et al., 2018; Enderle et al., 2015; Shurtleff et al., 2017). Whilst this may represent differences in the stringency of EV purification, it is notable that in two studies from the same group, Shurtleff et al. (2017) and Temoche‐Diaz et al. (2019), high levels of protection were seen for miRNA, but much lower for mRNA, suggesting that there may be distinct differences in localisation of RNA biotypes, which require careful investigation.

## 3 MECHANISMS OF RNA EV INCORPORATION

**FIGURE 2:**
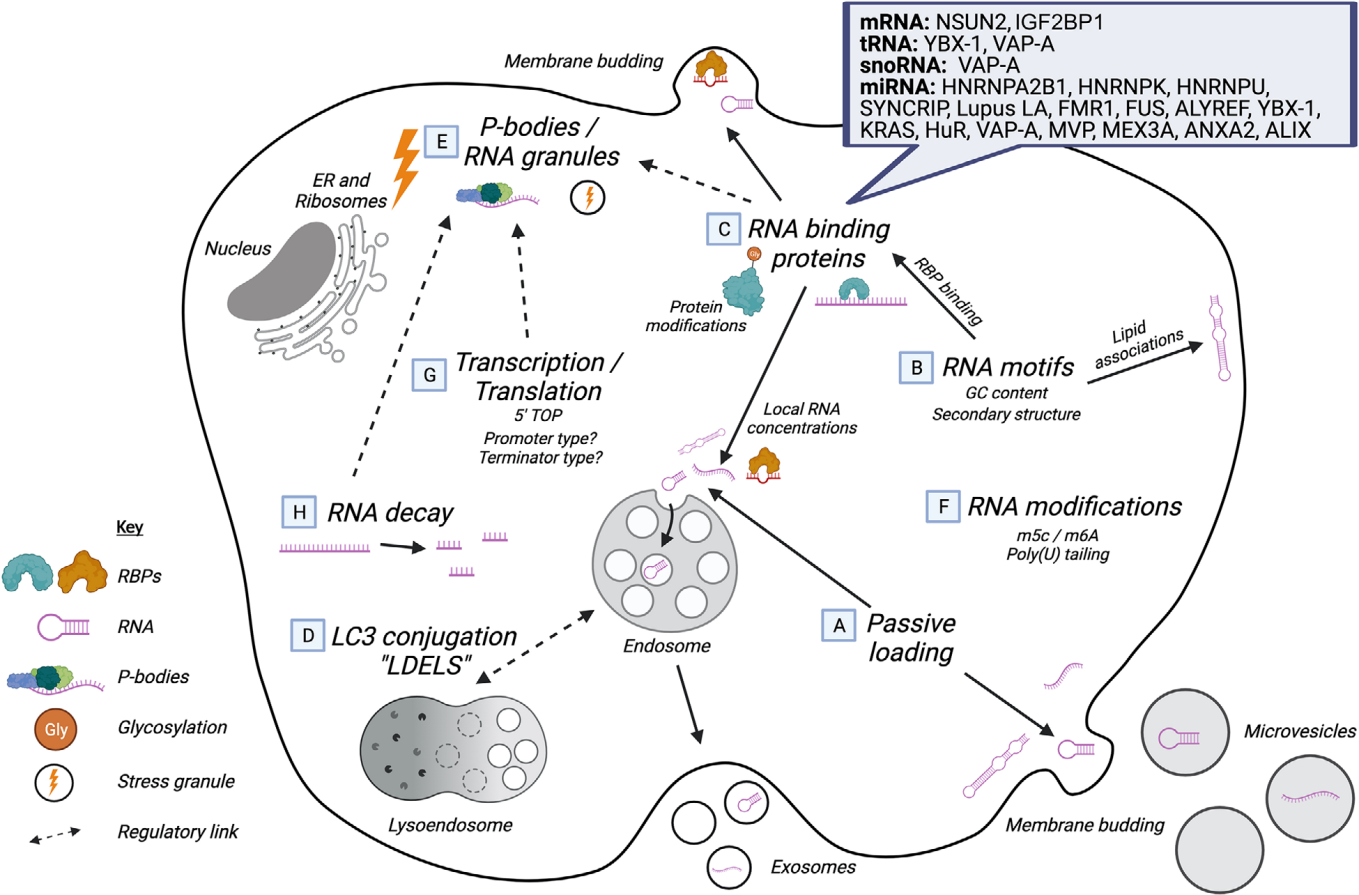
Mechanisms influencing RNA EV‐incorporation. Schematic diagram summarising examples of mechanisms which have been proposed to influence RNA EV‐incorporation. RNA content may be determined in a passive manner, purely dependent on the local RNA concentrations at EV generation sites at a given point in time (a) (Tosar et al., 2015). The GC content or secondary structure associated with RNA sequence motifs (b) may also alter the affinity of associations between RNA molecules and lipid membranes or RNA binding proteins (RBPs) (c), and thus alter RNA concentrations at sites of EV formation, at the multivesicular body or at the plasma membrane (Janas et al., 2020, 2012). Examples of these RBPs, and the RNA biotypes that they associate with, are highlighted in the blue box. RBP and RNA interactions can also be modulated by RBP post‐translational modifications, such as SUMOylation and glycosylation (H. Lee et al., 2019; Villarroya‐Beltri et al., 2013). More recently, regulatory links have been proposed between EV biogenesis and lysosomal autophagy, named LC3‐dependent extracellular vesicle loading and secretion (LDELS) (Leidal et al., 2020) (d). Many RBPs associated with EV loading (HNRNPA2B1, HNRNPA1, HNRNPU, IGF2BP1, SYNCRIP, YBX‐1, FMR1) are known components of stress granules or P‐bodies (e), suggesting a possible connection between these RNA granules and loading of EV‐RNA (Leidal & Debnath, 2020; Liu et al., 2021; Markmiller et al., 2018; Wolozin & Ivanov, 2019). Modification of RNA, for example, via poly‐uridylation (polyU), poly‐adenylation (polyA) or methylation, for example, 5‐methylcytosine (m^5^C) and N6‐methyladenosine (m^6^A), may also be involved in determining EV packaging or cell retention (Koppers‐Lalic et al., 2014) (f). Coupling of EV‐RNA loading and upstream processes such as transcription and translation may also influence EV RNA loading (g). For example, a high abundance of polymerase‐III‐encoded transcripts has been observed within EVs (Hardy et al., 2019; Lefebvre et al., 2016; Mosbach et al., 2021). Within the cell RNAs become degraded via RNA decay (g), with features such as RNA motifs and RNA modifications influencing this process, and may generate preferentially stable fragments that become loaded into EVs a means of disposal (Van Balkom et al., 2015)

Alongside the presence of this wide range of RNAs in EVs, many studies have identified differences in their relative abundance compared to the parental cells from which they derive. This has been argued as evidence towards specific, active mechanisms by which the RNA is packaged into EVs (Baglio et al., 2015; Cha et al., 2015; Chiou et al., 2018; Ekström et al., 2012; Villarroya‐Beltri et al., 2013). Such packaging processes have attracted great interest as they could be harnessed to facilitate the loading of RNA therapeutics, but also may give hints as to the physiological functions of EVs. A variety of processes, including RNA sequence motifs, RNA binding proteins, lipid interactions and RNA modifications, are under study as candidates for these mechanisms, with most work relating to miRNAs and mRNAs to date (Figure 2). Although differences in EV and cell RNA abundance may indicate that some species are actively loaded, Tosar et al. (2015) argue for a passive secretion model in their study of miRNAs, as 95% of 182 detected miRNAs were unaltered in EV relative to cell (Figure 2a). In further support of this model, the same group suggest that RNA stability is a key factor to maintaining high concentrations, which thus increase the chance of incorporation (Gámbaro et al., 2019). Nevertheless, despite the use of a high stringency four‐fold threshold for defining differential abundance, 5% of miRNAs were altered. The authors, therefore, suggest that, whilst intracellular RNA concentration is a major determinant of EV‐RNA abundance, specific secretion mechanisms likely also co‐exist alongside.

### 3.1 EV‐enriched RNA sequence motifs

One possibility for the determination of RNA packaging is the sequence composition of each RNA molecule. The existence of specific sequence motifs (Figure 2b) was first suggested in 2011, with identification of three motifs, ACCAGCCT, CAGTGAGC and TAATCCCA, enriched in EV‐mRNA of glioblastoma cells (Batagov et al., 2011; Skog et al., 2008). Additional work identified a 25 nt ‘zipcode’ motif (ACCCTGCCGCCTGGACTCCGCCTGT), present in the 20 most EV‐enriched transcripts in primary glioblastoma and melanoma cell lines, a motif which was further shown to mediate an increase in the level of eGFP in EVs of HEK293T cells (Bolukbasi et al., 2012). Both studies found these motifs to be present as part of predicted loop regions, indicating potential importance of structural features in the EV incorporation of the RNA. Bolukbasi et al. (2012) further suggest the binding of miR‐1289 to the zipcode is involved in loading, since the mRNA enrichment was influenced by miR‐1289 expression level. However, this same zip code sequence was not found to be enriched in EVs of the hepatic cell line MLP29, instead, C[TA]G[GC][AGT]G[CT]C[AT]GG[GA] was identified, leading the authors to propose that motif‐based systems are likely involved, but that they may be tissue‐specific (Szostak et al., 2014).

Studies of miRNA in EVs have also revealed enriched motifs. In miRNAs whose enrichment was common to both primary T‐cells and the Jurkat T‐cell line, in both resting and activated conditions, [GU]G[ACG][GC] (GGAG) and [CUG]CC[UGA] were found to be overrepresented in EV miRNAs, whilst several longer motifs were identified in cell miRNAs, one containing the 4 nt sequence UGCA (Villarroya‐Beltri et al., 2013). Mutation of GGAG to UGCA in candidate miRNAs induced an increase in EV/cell ratio, and a decrease changing from UGCA to GAGG, confirming the expected motif functionality. Several other studies have identified additional motifs associated with EV‐enriched miRNAs in other contexts, GGCU in murine hepatocyte 3A cells (Santangelo et al., 2016), UGGA in breast cancer MDA‐MB‐231 cells (Temoche‐Diaz et al., 2019), and AAUGC in miRNAs upregulated after inflammatory activation of monocytic THP‐1 cells (Wozniak et al., 2020). In a study of five cell types of different tissue origin, Garcia‐Martin et al. (2022) identify 4–7 nt motifs in each cell type which, although not directly compared, show differences from one another, indicating possible cell‐type‐specific loading. Interestingly, the authors find that these motifs are commonly GC‐rich, reminiscent of the C‐rich tendency seen in highly enriched miRNAs in colorectal cancer cell lines by Cha et al. (2015), where no specific motifs could be detected. Therefore, whilst specific motifs may have a role in RNA packaging in some contexts, they likely act in concert with a range of other factors.

One mechanism by which these motifs could act is to modulate direct RNA interaction with lipid membranes during biogenesis (Figure 2b). Czerniak and Saenz (2022) recently demonstrated that RNA oligomer binding is highly dependent on membrane fluidity, and thus differential lipid content. Indeed, earlier work by Janas et al. (2012) found that increased sphingomyelin/cholesterol content of liposomes (lipids enriched within raft regions in EVs), showed increased binding of tRNA^Sec^. This group also selected for RNA aptamers binding to liposomes containing lipid raft regions, identifying a set of motifs, UUGU, UCCC, CUCC and CCCU, which were common to a set of 4 nt motifs over‐represented in EV‐enriched pro‐tumoural miRNAs (Janas et al., 2020). Binding of the RNA to raft regions has been suggested to have greater secondary structure‐dependence, influenced by elements such as loop regions (Janas, 2006; Janas et al., 2012). Czerniak and Saenz (2022) also found that G‐rich G‐quadruplex structures showed increased binding to membranes in both a gel‐state, and in a more physiological fluid state. Together, these studies highlight a potential function of motifs to define specific RNA secondary structures and directly promote association with the MVB or plasma membrane, subsequently controlling loading into EVs.

### 3.2 EV RNA association with proteins

An alternative mechanism for activity of these motifs is that they mediate interaction with RNA binding proteins (RBPs) which then mediate the packaging process (Figure 2c). Villarroya‐Beltri et al. (2013) used biotin‐microRNA pull‐down with mass spectrometry to demonstrate binding of the RBPs hnRNPA2B1, hnRNPA1, hnRNPC, and RS4X to EV‐enriched miR‐198 but not cell‐enriched miR‐17. This was further confirmed for HNRNPA2B1 using electrophoretic mobility shift assays (EMSA). Additional miRNA motifs may act via binding to RBPs, Syncrip for GGCU (Santangelo et al., 2016), Lupus La for UGGA (Temoche‐Diaz et al., 2019), FMR1 for AAUGC (Wozniak et al., 2020) and Fus/Alyref for CGGGAG (Garcia‐Martin et al., 2022). Less follow‐up study has focused on mRNAs, although motifs identified by Batagov *et al*. were later shown by the same group to bind the methyltransferase NSUN2 and RBP YBX‐1 (Kossinova et al., 2017).

Motifs aside, a growing number of studies have identified additional proteins involved in EV loading, again primarily for miRNAs. KRAS (Cha et al., 2015), HuR (Mukherjee et al., 2016), MEX3C (Lu et al., 2017), Ago2 (Gibbings et al., 2009; Mckenzie et al., 2016), and IGF2BP1 (Ghoshal et al., 2019) have all been shown to have some effect on EV miRNA loading (Figure 2c). In an unbiased assessment of RBPs, Statello *et al*. carried out EMSA of EV protein incubated with either total EV RNA, cellular mRNA or cellular miRNA, identifying 90 interacting proteins; one (MVP) was functionally validated, while five others (HNRNPA2B1, HNRNPK, HNRNPU, NSUN2 and VAP‐A) were demonstrated to have involvement in independent studies (Barman et al., 2020; Kossinova et al., 2017; Leidal et al., 2020; Villarroya‐Beltri et al., 2013; Zietzer et al., 2020). On the other hand, in 2019 it was reported that many of these RBPs, including hnRNPA2B1 and MVP, were not associated with EVs, but were co‐isolated contaminants, highlighting the importance of defined EV populations (Jeppesen et al., 2019). Other biotypes of RNA have remained largely un‐investigated; however, recent work has shown that VAP‐A has an effect on snoRNA and tRNA loading as well as miRNA (Barman et al., 2020). Of notable interest, YBX‐1 is the only RBP implicated in both short (miRNA, tRNA, vault RNA, Y‐RNA) and long (mRNA) RNA loading (Kossinova et al., 2017; Shurtleff et al., 2016, 2017). Contrastingly, the effect of KRAS was seen in miRNA but not long RNA (Cha et al., 2015; Hinger et al., 2018). Therefore, it seems that whilst protein‐mediated loading is a common process for multiple RNA biotypes, there may be little overlap in the specific proteins involved.

Although proteins have clear involvement in the packaging of RNA into EVs, there are many possible pathways by which they could mediate loading. It is possible that they directly transport the RNA to sites of EV‐biogenesis (the plasma membrane for MVs or the MVB for exosomes), or that they act indirectly by driving asymmetry in RNA subcellular localisation (Tosar et al., 2021). Many of the RBPs that have been identified as involved in RNA packaging (HNRNPA2B1, HNRNPA1, YBX‐1, SYNCRIP and HNRNPU) were found to interact directly with the tetraspanin CD81, or two of its interacting proteins, ICAM‐1 and EWl‐2 (Perez‐Hernandez et al., 2013). Cellular RNA distribution is also highly assymetric (Benoit Bouvrette et al., 2018; Khong et al., 2017; Matheny et al., 2019), and recent studies have indicated the possiblity that RNA loading of EVs is linked to organellar structures such as the nucleus (Lässer et al., 2017) and endoplasmic reticulum (Barman et al., 2020). Leidal et al. (2020) also identified a striking 81% overlap between the protein interactome of the autophagy component phospholipid‐conjugated LC3 and the EV proteome, with a heavy enrichment for RBPs. Knockout of ATG7 and ATG12, essential for LC3 conjugation but not degradative autophagy, also had significant impact on both EV protein and EV small RNA content (miRNA and snoRNA). This led the authors to propose LC3‐dependent extracellular vesicle loading and secretion (LDELS) as a mechanism for cargo loading, evidencing at least a regulatory linkage between EV biogenesis and lysosomal autophagy (Figure 2d). Also, noted in the study was the presence of a large number of RBPs known to be present in stress granules and P‐bodies, hinting at a possible connection between these RNA granules and loading of EV‐RNA (Figure 2e) (Leidal & Debnath, 2020). In HEK293 cells, Liu et al. (2021) recently found an 18.4% overlap of the P‐body proteome, and a 28.7% overlap of the stress granule proteome, with small EVs_._ The authors further propose that phase separation of RBPs such as YBX‐1, and bound RNAs into these RNA granules, acts in concentrative capture of these components and subsequent sorting into the MVB. Future work to directly compare the RNA content of EVs to sub‐cellular fractions may therefore be informative in pinpointing where, and thus how, RNA packaging is occuring. Additionally, consideration of the heterogeneity of EVs is of critical importance. Barman et al. (2020) found that in small EVs a dense subpopulation, constituting 10% of the total EV population, contained around nine‐fold more RNA per EV than the light fraction. Given EV sub‐types deriving from different sub‐cellular locations likely contain different RNA populations, study of RNA loading processes need account for this.

### 3.3 Influence of RNA and protein modifications on their EV incorporation

Proteins may undergo an array of chemical post‐translational modifications (PTMs), the most well known of which include phosphorylation, glycosylation, methylation and ubiquitination. This is one possible mechanism by which RNA and RBP interaction with EV biogenesis components could be regulated (Carnino et al., 2020). Phosphorylation of YBX‐1 was seen to be higher in HEK293 cell extract but not EVs, suggesting that dephosphorylation may be a mechanism by which the protein, and bound mRNAs, may be directed towards EVs (Kossinova et al., 2017). SUMOylation (small ubiquitin‐like modification) of hnRNPA2B1 has also been shown to be specific to EV‐localised protein, whilst anacardic acid inhibition of SUMOylation decreased miR‐198 levels in the EV (Villarroya‐Beltri et al., 2013). O‐GlcNAc glycosylation of hnRNPA2B1 has also been found to alter EV miRNA content, a modification which is regulated by oxidative stress‐induced phosphorylation of Caveolin‐1 (H. Lee et al., 2019). Since many of these PTMs, such as phosphorylation, are rapidly reversible, they likely play a wider role in regulating RNA loading of EVs under different cellular conditions.

Another potential mechanism for the control of EV RNA incorporation, with or without protein involvement, is specific modifications, or additions, to the RNA itself (Figure 2f). Despite an overall underrepresentation of miRNA in EVs, poly‐uridylated miRNAs were shown to be significantly enriched, and poly‐adenylated miRNAs significantly depleted, indicating that non‐templated additions may be an important determinant of specific secretion (Koppers‐Lalic et al., 2014). An RNA modification, although not identified, has also been proposed as a mechanism for specific incorporation of tRNA (Shurtleff et al., 2017). It is likewise intriguing that NSUN2, identified by both Statello *et al*. and Kossinova *et al*. as binding mRNA, functions as a methyltransferase to add 5‐methylcytosine (m^5^C) modifications to multiple RNAs, whilst ALYREF (miRNA loading; Garcia‐Martin et al., 2022) and YBX‐1 (miRNA, tRNA, vRNA, YRNA loading; Batagov et al., 2011; Kossinova et al., 2017; Shurtleff et al., 2016, 2017) function as m^5^C readers (Yang et al., 2017; Zou et al., 2020), hinting at the potential wider importance of the m^5^C modification, perhaps linked to translation or RNA stability (Henry et al., 2020; Yang et al., 2017). A recent study has also demonstrated that the N6‐Methyladenosine (m6A) modification is highly enriched in small and long RNAs associated with EVs from plant apoplastic fluid, alongside highly abundant circRNAs (Zand Karimi et al., 2022). Given the N6‐Methyladenosine (m6A) modification is known to be involved in the formation, stability and export of circRNAs (X. Huang et al., 2022), this may represent an important loading determinant for further investigation. However, it must be noted that m6A enrichment was most prominent in non‐vesicular extracellular RNA, again highlighting a need for careful differentiation of vesicular and non‐vesicular RNAs (Zand Karimi et al., 2022).

In 2021, Flynn et al. (2021) demonstrated for the first time that RNAs can be N‐glycan‐modified. These ‘glycoRNAs’ were found to be primarily associated with the surface of membrane‐bound organelles and trafficked to the cell surface where they could interact with sialic acid binding‐immunoglobulin lectin‐type receptors. Whilst the glycoRNAs identified by Flynn *et al*. were exclusively small RNAs (including Y RNAs, snRNAs, snoRNAs and tRNAs), membrane‐bound lncRNAs were also identified by N. Huang et al. (2020). Together these studies provide an intriguing possibility that RNAs could be displayed on the surface of EVs in a similar manner.

### 3.4 Transcription and translation‐coupled loading

Several studies have noted the high abundance of polymerase‐III‐encoded transcripts in small EV samples, including vault RNAs, Y‐RNAs and 7SL RNA (Hardy et al., 2019; Lefebvre et al., 2016). Recent work by Mosbach et al. (2021) demonstrated that a construct with a U6 snRNA‐derived polymerase III promoter and U6 terminator (polyT) was more efficiently secreted into EVs than the same construct with a CMV‐polymerase II promoter with either poly(A) or U1‐3′ box terminator. This could be driven by the increased cellular abundance seen by the authors, but could also be driven by coupling of EV‐RNA loading to more upstream processes such as transcription and translation (Figure 2g). For example, it is interesting to note that the error rate of polymerase III (in yeast) is higher than that of polymerase I or polymerase II (Gout et al., 2017), raising the possibility that products of polymerase III transcription are more likely to be targeted for degradation, in line with the waste disposal function of EVs (Figure 2h). Given also the association of poly (U) with EV‐RNA (Hardy et al., 2019; Koppers‐Lalic et al., 2014), the findings by Mosbach et al. (2021) may also be due to the presence of the U6 terminator sequence. mRNAs containing 5′ TOP motifs, which function in coupling their translation to mTORC1 metabolic regulator complex, have also been found to be highly abundant in EVs (Shurtleff et al., 2017). Intriguingly, translational inhibition of HEK293 cells with cycloheximide, which blocks P‐body assembly, also reduced EV numbers and YBX‐1 mediated EV secretion of miR‐223 (Liu et al., 2021). Further work is therefore needed to elucidate whether factors associated with RNA transcription and translation relate to their EV incorporation, as may further aid design of constructs for efficient loading of customised cargoes.

## 4 EVS AS A MECHANISM FOR RNA DISPOSAL

**FIGURE 3:**
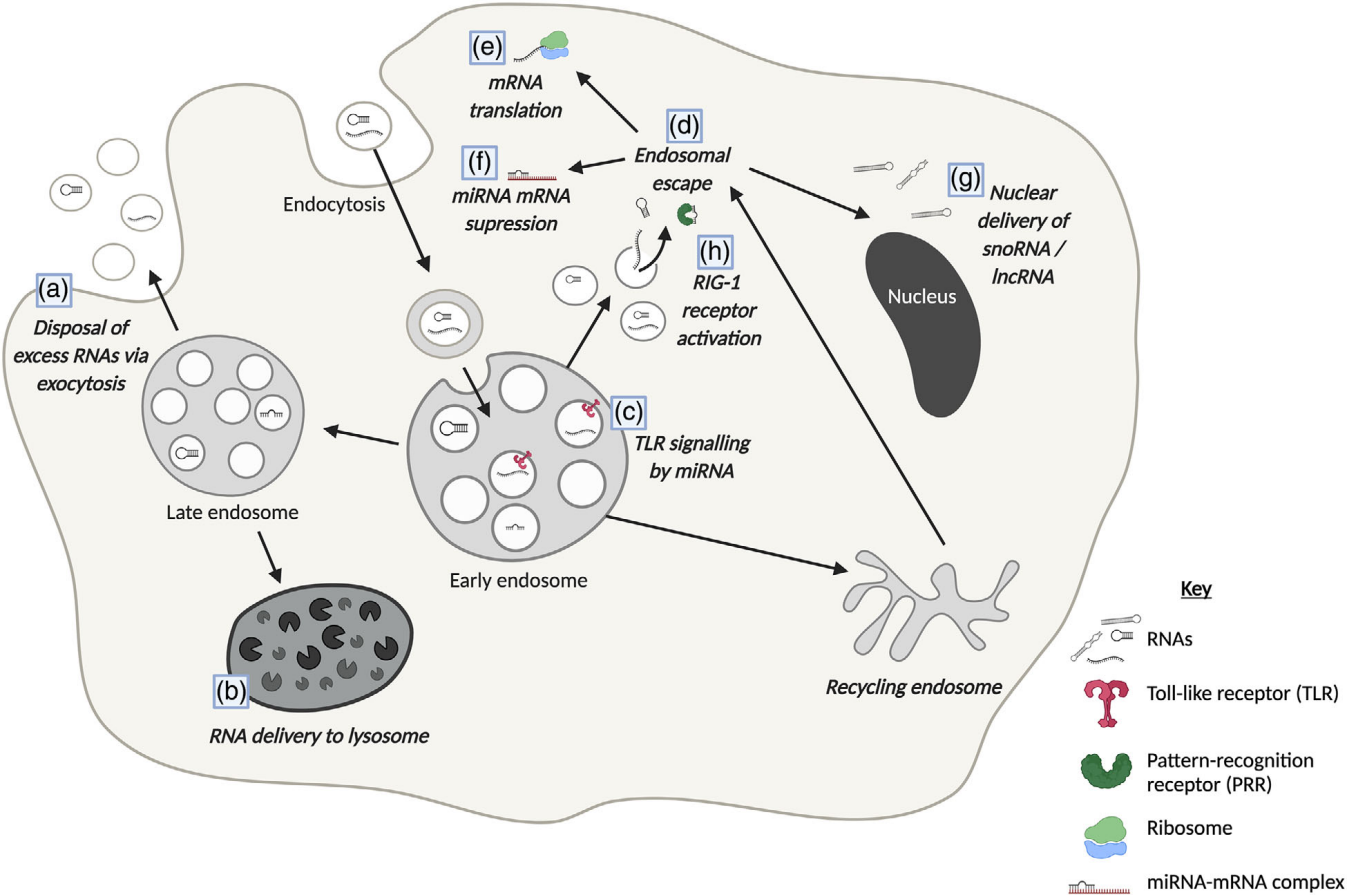
Functions of EV‐RNA. Schematic diagram to summarise the diversity of known mechanisms by which EV‐associated RNAs can modulate cellular activity. Exocytosis from EV‐producing cells may facilitate the disposal of excess or aberrant RNAs so as to not disrupt cell signalling pathways (a). EVs may also be internalised into recipient cells via endocytosis subsequently fusing with the endosome. EV RNAs present within the endosomal pathway may ultimately be degraded via the lysosome, as a way to outsource degradation to these recipient cells (Desdín‐Micó & Mittelbrunn, 2017) (b) RNA has also been shown to activate toll‐like receptors (TLRs) from within endosomes (c), for example, miR‐21 and miR‐29a activation of *TLR‐7* and *TLR‐8*a (Fabbri et al., 2012). Endosomal escape has been suggested to be a limiting factor for functional cargo delivery (d), with only 10%–30% of internalised EVs estimated to release their cargo to the cytoplasm (Bonsergent et al., 2021; Joshi et al., 2020). Once RNAs have achieved endosomal escape, they can elicit effects from within the cytoplasm, such as translation of mRNA (Albanese et al., 2021; Valadi et al., 2007) (e), miRNA‐induced translational repression of mRNA (f), nuclear delivery (Hinger et al., 2018; Rimer et al., 2018) (g), or receptor activation, such as the pattern‐recognition receptor RIG‐1 (h) (Nabet et al., 2017)

Given the evidence for the regulated loading of RNA into EVs, this implies that the process has a specific function to the cells. However, it raises the question as to whether it is the *removal* of that RNA from the producing cell, or the *recognition* or *delivery* of the RNA to any recipient cells that is of primary physiological relevance (Figure 3). EVs may have a role as a homeostatic mechanism for the producing cell, as a means for disposing of unwanted cellular material (Desdín‐Micó & Mittelbrunn, 2017). Alongside early evidence for a function in removal of reticulocyte transferrin receptor (Harding et al., 1984; Pan & Johnstone, 1983), they have been implicated in clearance of toxic misfolded proteins in neurodegenerative diseases (Emmanouilidou et al., 2010; Guo et al., Hill, 2015; Yuyama et al., 2012), removal of cholesterol to relieve lysosomal dysfunction in Niemann–Pick type C1 disease (Strauss et al., 2010) and elimination of cytosolic‐localised nuclear DNA fragments to avoid activation of DNA damage response (A. Takahashi et al., 2017). For RNA, EVs have also been proposed as a means to dispose of miRNAs present in excess to their target mRNAs, since overexpression of the mRNAs resulted in EV depletion of regulatory miRNAs, whilst overexpression of the miRNAs themselves resulted in enrichment in EVs (Figure 3a) (Squadrito et al., 2014). Similarly, loading of 5′ tRNA halves has been suggested as a means of releasing fragments that would otherwise induce T‐cell activation (Chiou et al., 2018), whilst the abundance of Y‐RNA and mRNA fragments has been suggested as indicative of removal of aberrant RNA (Van Balkom et al., 2015). Of particular interest, an expanded CAG repeat motif within *huntingtin* mRNA showed an increase in EV incorporation compared to normal *huntingtin* in mouse striatal neurons (X. Zhang et al., 2016). Finally, enrichment of circRNAs in EVs has been suggested to be a mechanism for clearance of circRNAs which are resistant to degradation by exonucleases (Lasda & Parker, 2016). Whilst release of some of these molecules may have detrimental impacts on recipient cells, it may be that this is a means by which an overloaded cell outsources degradation to healthy neighbouring cells (Figure 3b) (Vidal, 2019).

## 5 MECHANISMS OF EV‐MEDIATED RNA DELIVERY AND FUNCTION IN RECIPIENT CELLS

**FIGURE 4:**
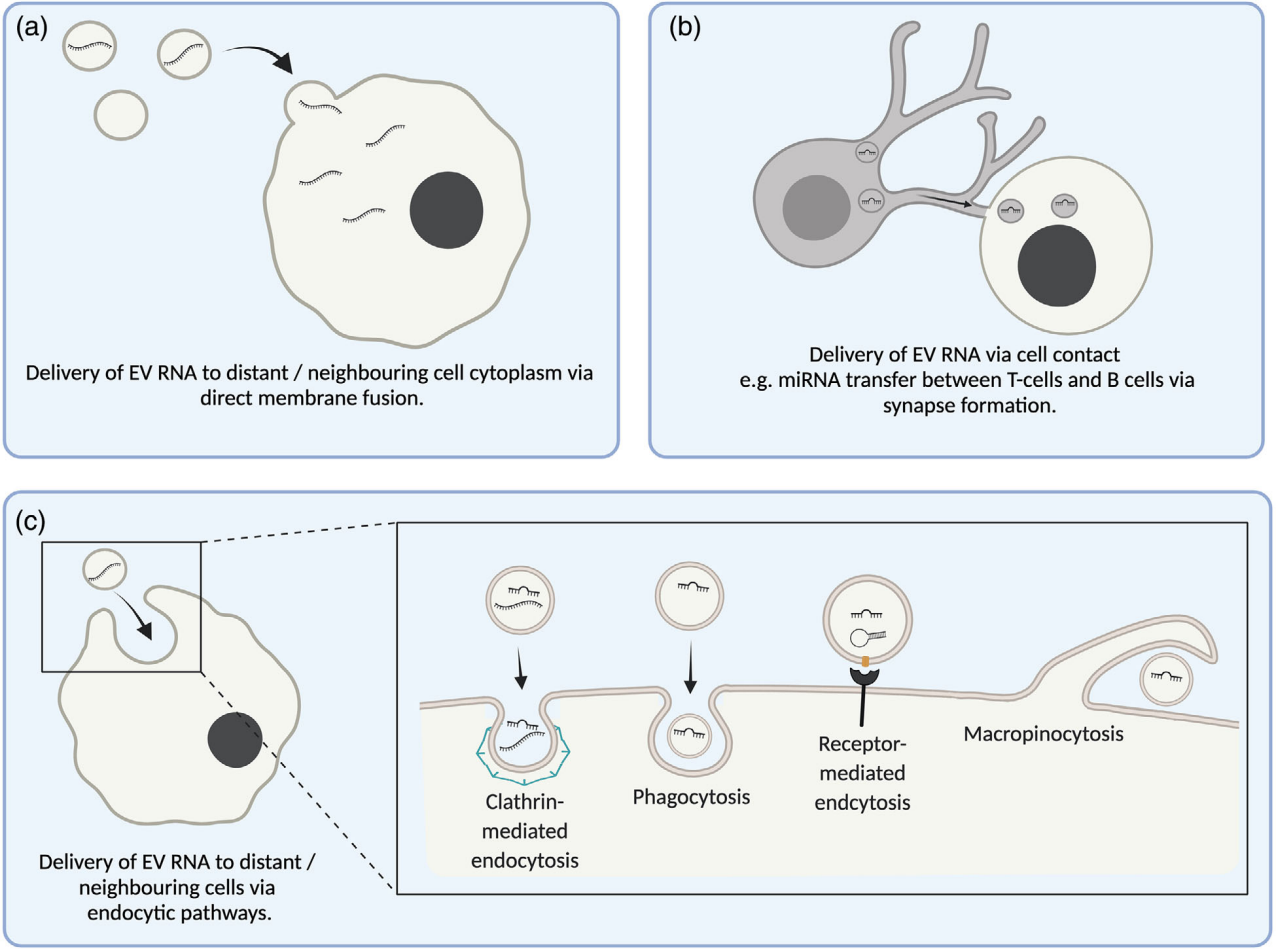
Mechanisms of EV uptake. Delivery of EV RNAs to the recipient cell can occur via direct membrane fusion between the EV and the cell membrane of the recipient cell (a). An alternative EV uptake mechanism is via cellular contacts between EV producing and recipient cells, for example via nanotube structures or synapse formation (Haimovich et al., 2017; Mittelbrunn et al., 2011) (b). A common EV uptake mechanism is endocytosis (c), including clathrin‐mediated, phagocytosis, receptor‐mediated and micropinocytosis (Mulcahy et al., 2014)

Perhaps the greatest interest in EV RNAs, however, has been their potential role in driving phenotypic effects in recipient cells through cytoplasmic delivery. Three main mechanisms for EV‐RNA uptake have been suggested; firstly, cell surface membrane fusion, which may be mechanistically similar to viral membrane fusion, and would imply direct delivery of cargo to the cytoplasm (Figure 4a) (Montecalvo et al., 2012; Parolini et al., 2009). Secondly, EVs may be transferred via cell‐contact dependent mechanisms (Figure 4b). In 2011, miRNA transfer between donor T‐cells and recipient B cells was only observed when synapse formation was induced, but blocked by neutral sphingomyelinase inhibition or *Rab27a* knockdown, indicating both EV and cell‐contact dependence (Mittelbrunn et al., 2011). mRNA transfer was also found to be contact‐dependent in a variety of cell lines by Haimovich et al. (2017), with the authors concluding transfer occurred only via nanotube structures. However, the majority of EV uptake is believed to occur via endocytic pathways, which include clathrin‐mediated, caveolin‐dependent and receptor‐mediated endocytosis, micropinocytosis, and phagocytosis (Figure 4c) (Mulcahy et al., 2014). These mechanisms imply that the cargo would be delivered to the endosome. Some EV‐RNAs have been found to trigger signalling pathways from within the endosomal compartment (Figure 3c) (Fabbri et al., 2012; Moroishi et al., 2016), and indeed since single‐stranded RNA can activate toll‐like receptors in endosomes (Diebold et al., 2004; Karikó et al., 2004), it is possible that the RNA fragments present in EVs could function in a similar manner. However, cytoplasmic cargo delivery would require endosomal escape to avoid lysosomal degradation, and it is this endosomal escape process that has been suggested by many to be a key limiting factor (Figure 3d) (Bonsergent et al., 2021; Heath et al., 2019; Hung & Leonard, 2016; Kanada et al., 2015). Using membrane‐bound GFP/NanoLuc tagged‐CD63 or luminal NanoLuc‐tagged Hsp70 to assess cytoplasmic delivery, Joshi et al. (2020) and Bonsergent et al. (2021) found between 10% and 30% of internalised EVs to release cargo to the cytoplasm, although with a higher rate at a longer timepoint. Treatment with different chemical compounds also has an impact on protein delivery, with an increase seen when recipient cells were treated with chloroquine or UNC10217832A (to promote endosomal lysis) or concanomycin A (to inhibit lysosomal acidification), and a reduction when treated with Bafilomycin A1 (to inhibit endosomal acidification), indicating that endosomal acidification is of key importance to cargo delivery (Bonsergent et al., 2021; Heath et al., 2019; Joshi et al., 2020; Kanada et al., 2015). Stress treatments have also been suggested to increase delivery via EVs, with irradiation of recipient cells shown to increase EV uptake (Mutschelknaus et al., 2016), and mRNA delivery increased by multiple different stress treatments (heat shock, hydrogen peroxide, dithiothreitol or serum starvation) (Haimovich et al., 2017). Given that in vivo tools developed to report EV‐mediated communication displayed increased reporter signalling during chronic inflammation or myocardial infarction (Das et al., 2019; Ridder et al., 2014), the healthy functioning of the endolysosomal system of the recipient cell may be critical to cytoplasmic delivery of EV‐RNA. This perhaps provides an explanation to the variable conclusions on RNA transfer that have been seen in other studies, but also indicating that in pathological scenarios where endolysosomal function may be particularly impaired (such as in neurodegenerative disease), delivery could be significantly altered.

### 5.1 mRNA translation

Whilst seminal work by Valadi et al. (2007) found that mRNAs from mouse EVs could be translated in human mast cell recipients (Figure 3e), a lack of tools to effectively study this process has made it a highly challenging task to provide direct evidence that specific mRNA cargoes are responsible for specific responses (Mateescu et al., 2017; Somiya, 2020). However, multiple reporter systems have now been developed to study EV‐mRNA function, both in vitro and in vivo. Several groups have made use of Cre‐Lox reporter systems in which Cre mRNA is expressed in one cell line, organism, or tissue, and subsequently transferred via EVs to a recipient, where it is translated and results in the excision of LoxP sites in a reporter construct, thus permitting the expression of a detectable protein such as gfp, rfp, luciferase or beta‐galactosidase. Such a system was first used to demonstrate that haemopoietic‐derived, RNAse treated, EVs, injected into the cerebellum, could mediate beta‐galactosidase reporter activation in a range of cell types including Purkinje neurons and microglial cells, which were not induced by the injection of purified protein or cell lysate (Ridder et al., 2014). Further work by the same group demonstrated that transplanted TU2449 astrocytic or LLC2 cells, or purified EVs, could also mediate reporter activation (Ridder et al., 2015). A similar reporter, with Cre inducing a switch from dsRed to eGFP expression, showed activation both in vitro, in co‐culture and transwell experiments, and in vivo, with injected EVs, in a range of tissues including lymph nodes, lungs and spleen (Zomer et al., 2015). However, this activity cannot be unequivocally attributed to Cre mRNA, since the Western blotting or ELISA techniques used to demonstrate absence of Cre protein have lower sensitivity than the qPCR used to detect mRNA (Ridder et al., 2014, 2015; Zomer et al., 2015). In a luciferase‐based reporter in HEK‐293F cells, the presence of full‐length mRNA in both small and large EVs was also demonstrated; however, using transcriptional and translational inhibitors it was found that no reporter activation could be attributed specifically to the mRNA (Kanada et al., 2015). Similarly, using a NanoLuc assay combined with a CRISPR‐Cas13b‐based RNA editing tool, Somiya and Kuroda (2022) show reporter signal to be due to protein rather than mRNA transfer in HEK293T cells. However, when EV‐producing cells were transfected with vesicular stomatitis virus G (VSVG) plasmids, which incorporate into the EV membrane and promote fusion with recipient cells, mRNA transfer *could* be confirmed, in two separate studies (Albanese et al., 2021; Somiya and Kuroda, 2022). In vivo, Cre recombination in the reporter system developed by Ridder et al. was also dramatically higher in the context of chronic inflammation or myocardial infarction (Das et al., 2019; Ridder et al., 2014). Together these studies demonstrate that whilst functional transfer of mRNA is possible, much more work is needed to understand what specific circumstances it does occur in. In particular, in vitro systems may not adequately mimic the more complex in vivo environment, highlighting the need for more adaptation of these elegant reporters to in vivo model systems.

### 5.2 miRNA function

Alongside these reporter systems to study delivery of mRNA, de Jong et al. (2020) have also developed a complementary CRISPR‐Cas9‐based method to study transfer of small RNAs. The authors found that the functional transfer of the RNA differed depending on the combination of donor and receptor cells used (notably with no transfer when using HEK293 cell donors), although with the percentage of reporter positive cells reaching a maximum of 0.7%. This indicates that small RNA delivery is a process that *can* occur; indeed, several studies have begun to harness it for therapeutic purposes. EV‐delivered miRNAs can function in mRNA suppression (Figure 3f), with miR‐21 show to mediate glioblastoma‐associated microglial reprogramming in a therapeutic context (Abels et al., 2019), whilst synthetic siRNA was also shown to supress oncogenic KRAS and tumour growth (Kamerkar et al., 2017). However, this therapeutic application is currently limited by incomplete understanding of the underlying EV biology (Melling et al., 2019), and given the low percentages of effective transfer, one can question whether the process occurs at a sufficient rate under *normal* physiological conditions to be biologically relevant.

A key question to understanding this biological relevance is how many RNA molecules are delivered in these systems. Chevillet et al. (2014) estimate an average abundance of one copy of any miRNA per 121 EVs (ranging from 1 per 9 to 1 per 47,162 for specific miRNAs), similar to an estimate of 1 in 100 by Wei et al. (2017). In order to elicit a canonical suppressive effect on mRNA, it is thought that around 100 miRNA copies of per cell are needed as a threshold minimum (Brown et al., 2007) which, for the average miRNA would require delivery of the cargo of 11,000 EVs if the miRNA is absent in the recipient cell. However, given the evidence for a high degree of heterogeneity in the quantity of RNA between EV subpopulations, this might vary widely (Barman et al., 2020). Furthermore, lower miRNA copy numbers would be required to elicit an effect for a miRNA already close to the threshold in the receiving cell—thus it is feasible that just a few miRNAs from EVs could act to tip the balance. Aside from this canonical mechanism, it is also possible that the function of the miRNA in a different manner, for example, that bulk EV miRNA delivery mediates a non‐specific effect, by effectively ‘drowning out’ low abundance miRNAs in the recipient cells, so that they can no longer compete for binding to the RISC complex, an effect seen previously for shRNA (Brown et al., 2007; Grimm et al., 2006). However, such a mechanism of action would not be detected by current reporter systems, leaving scope for exploration of new avenues by which EV‐RNAs could maintain relevant functionality.

### 5.3 Function of other RNA biotypes

Delivery of other RNA biotypes beyond the more well‐known mRNA and miRNA species has also begun to be investigated. The tRNA^Gly^_GCC_, or 5′‐tRNA‐half, has been identified as particularly abundant in EVs and has undergone most functional study (Baglio et al., 2015; Cooke et al., 2019; Gámbaro et al., 2019). Cooke et al. (2019) show that direct treatment of fibroblasts with this 5′‐tRNA‐half significantly reduced global protein synthesis, whilst Gámbaro et al. (2019) demonstrate delivery of a synthetic form using fluorescence microscopy and stem‐loop‐RT‐qPCR, together indicating that, if delivered in high enough quantities, EV‐tRNA has the potential to induce large phenotypic effects. Delivery of lncRNA (oncogenic *CRNDE)* and snoRNA (Rpl13a‐intron derived snoRNA U33) to the nuclei of recipient cells has been observed (Figure 3g), using a CRISPR‐Display luciferase reporter system and direct labelling in transwell co‐cultures respectively (Hinger et al., 2018; Rimer et al., 2018). Rimer et al. (2018) further demonstrate, in a long‐range transfer model using shared circulation of wildtype and Rpl13a knockout mice, significant differences in ribosomal RNA 2′‐O‐methylribosylation, indicating functioning of the snoRNA in recipient cells. The non‐coding *7SL* RNA, notably highly abundant in both human and *Drosophila* EVs (Lefebvre et al., 2016), has also been demonstrated to activate the pattern‐recognition receptor RIG‐1 in the cytoplasm of stromal cells, leading to an increased inflammatory response (Figure 3h) (Nabet et al., 2017). Interestingly similar pro‐inflammatory activity of EV miRNAs, miR‐21 and miR‐29a, was observed by Fabbri et al. (2012), but via the endosomal toll‐like receptors *TLR‐7* and *TLR‐8*. Induction of toll‐like receptor signalling by EVs was also seen by Moroishi et al. (2016), and strongly increased by knockout of LATS1/2, which the authors attribute to a dramatic increase in RNA cargo of the EVs. Together these studies indicate that EV‐associated RNA has an important role in inflammatory signalling pathways.

Moving beyond the known RNA species, however, intact RNA species may not be the most biologically relevant, simply by way of abundance. For example, Wei et al. (2017) estimate the most abundant single mRNA to be present at 1 per 1000 EVs, *any* mRNA molecule at 1 per 10 EVs, but exonic or intronic mRNA fragments at 1 per EV. rRNA, repeat region and snRNA fragments had an abundance higher still, so these fragmented species would plausibly require uptake and delivery from fewer EVs to have an effect. Indeed, since single‐stranded RNA can activate toll‐like receptors in endosomes (Diebold et al., 2004; Karikó et al., 2004), it is possible that EVs mediate delivery of fragmented RNAs of a range of biotypes that could act to stimulate inflammatory signalling. An increasing body of evidence also indicates the importance of rRNA fragments in controlling cell proliferation, apoptosis, stress and DNA‐damage response, so investigating these higher abundance fragmented species would be an interesting future avenue of research (Z. Chen et al., 2017; Cherlin et al., 2020; Ding et al., 2016; H.‐C. Lee et al., 2009; Zhu et al., 2018).

## 6 CONCLUSION

The mRNA and miRNA content of EVs has been extensively studied since its discovery in 2007, but accumulating data clearly demonstrates the presence of a much broader range of RNA species which are just beginning to be investigated. Whilst some key mechanisms of packaging of these RNAs have begun to be elucidated, it seems that multiple factors likely act cumulatively to encourage loading, with a greater understanding of these factors enabling the design of customised cargoes for therapeutic use. In addition, since the RNA species present in EVs are likely as heterogeneous as EVs themselves, defining which EV subpopulations contain which RNAs, and how this links to EV biogenesis, is essential to understanding their physiological functions. Furthermore, this work must extend to the study of non‐physiological scenarios to understand alterations occurring in pathological conditions. Whilst much work has focused on the translatability of mRNAs in recipient cells, investigating function of other RNA biotypes is needed, and will be aided by increasingly sophisticated tools to label EVs and their cargo, to study EV biogenesis and uptake both in vitro and in vivo.

## CONFLICTS OF INTEREST

The authors declare no conflicts of interest.

## AUTHOR CONTRIBUTIONS

Elizabeth R. Dellar and Claire Hill researched the content of the article, Elizabeth R. Dellar wrote the manuscript and Claire Hill produced figures. David R.F Carter, Luis Alberto Baena‐Lopez, Claire Hill and Genevieve EMelling contributed to discussion and provided critical editorial input. All authors reviewed the final article before submission.
